# Lack of guilt, guilt, and shame: a multi-informant study on the relations between self-conscious emotions and psychopathology in clinically referred children and adolescents

**DOI:** 10.1007/s00787-015-0749-6

**Published:** 2015-07-29

**Authors:** Peter Muris, Cor Meesters, Jolina Heijmans, Sandra van Hulten, Linsy Kaanen, Birgit Oerlemans, Tessa Stikkelbroeck, Tim Tielemans

**Affiliations:** Maastricht University, Maastricht, The Netherlands; Virenze-RIAGG, Maastricht, The Netherlands; Stellenbosch University, Stellenbosch, South Africa; Clinical Psychological Science, Faculty of Psychology and Neuroscience, Maastricht University, P.O. Box 616, 6200 MD Maastricht, The Netherlands

**Keywords:** Self-conscious emotions, Guilt and shame, Psychopathology, Children and adolescents, Achenbach System of Empirically Based Assessment

## Abstract

The present study examined the relationships between dysregulations in self-conscious emotions and psychopathology in clinically referred children and adolescents. For this purpose, parent-, teacher-, and self-report Achenbach System of Empirically Based Assessment data of 1000 youth aged 4–18 years was analyzed as this instrument not only provides information on the intensity levels of lack of guilt, guilt, and shame, but also on the severity of various types of psychopathology. The results first of all indicated that dysregulations of self-conscious emotions were more common in this clinical sample than in the general population. Further, a consistent pattern was found with regard to the relationships between self-conscious emotions and childhood psychopathology. That is, lack of guilt was predominantly associated with oppositional defiant and conduct (i.e., externalizing) problems, while guilt and shame were primarily linked with affective and anxiety (i.e., internalizing) problems. By and large, these findings confirm what has been found in non-clinical youth, and suggest that self-conscious emotions play a small but significant role in the psychopathology of children and adolescents.

## Introduction

The self-conscious emotions of guilt and shame help people to navigate successfully in the social environment. That is, when displaying inappropriate or bad behavior in the real or imagined presence of others, the prototypical feelings of remorse and regret prompt the individual to engage in compensatory interpersonal behaviors [1]. Guilt, which is more concerned with the negative evaluation of a specific behavior (“I did *that* wrong”), motivates the person to engage in reparative behavior by making apologies and engaging in attempts to fix the situation, while shame, which is typically characterized by a negative evaluation of the self (“*I* did that wrong”), leads to submission and avoidance and sometimes hostility and retaliation [2, 3]. On the basis of these descriptions, one might get the impression that guilt is somewhat more adaptive than shame, but it is good to keep in mind that in essence both types of self-conscious emotions have their own functionality in correcting moral and social transgressions. However, this is no longer true when the emotions of guilt and shame become seriously dysregulated. When feelings of guilt and shame are too easily and too frequently triggered, the compensatory interpersonal behaviors may become too dominant and start to interfere with the person’s daily functioning. Or otherwise, when feelings of guilt and shame are not or insufficiently elicited, transgressions will not be followed by corrective behaviors and likely cause interpersonal problems.

In the adult literature, clear support can be found for the notion that the dysregulation of shame is associated with various types of psychopathology. That is, various studies have demonstrated that high levels of shame are associated with a variety of psychological symptoms including anger and aggression [4], depression [5], post-traumatic stress disorder [6], anxiety disorders [7], eating problems [8], personality pathology [9], suicidal and self-injurious behavior [10], and substance abuse [11]. The relation between guilt and psychopathology is more ambivalent, but the general idea is that high levels of this self-conscious emotion are also maladaptive, in particular when it is experienced in an obsessive, ruminative way or fused with feelings of shame [1]. This is preeminently true in the case of depression for which “inappropriate guilt” is even considered as one of the key symptoms defining the disorder [12, p. 161]. Further, it has been argued that extremely low levels of guilt can also be problematic as this may hinder the development of empathy and conscience [13, 14]. Indeed, “lack of remorse or guilt” has been consistently associated with aggressive and antisocial behavior [15], and is currently added as a specifier to the diagnostic criteria of conduct disorder [12] for which it has been shown to predict a negative prognosis [16].

Research on the relation between guilt and shame and psychological symptoms in children and adolescents is sparser. A recent review by Muris and Meesters [2] identified 22 studies exploring the relation between these self-conscious emotions and psychopathology in youths, and since then a special issue has appeared in *Child Psychiatry and Human Development* including four new investigations on the link between guilt and/or shame and trauma-related problems [17], internalizing problems [18], anxiety symptoms [19], and risky and illegal behaviors [20] in young people. Altogether, findings are largely in line with what has been found in adult populations. That is, data generally indicate that shame is positively associated with internalizing as well as externalizing symptoms of children and adolescents [2]. The results for guilt have been more ambiguous. On the one hand, there is research demonstrating that guilt protects youth against the development of externalizing problems [21], and showing that extremely low levels—and especially lack—of guilt are associated with the occurrence of externalizing problems [4, 15]. On the other hand, studies can be found indicating that high levels of this self-conscious emotion, especially when contaminated with shame, are accompanied with higher levels of internalizing symptoms [17, 22].

One obvious shortcoming of the research conducted so far on the relation between self-conscious emotions and psychopathology in youth pertains to its almost exclusive reliance on non-clinical samples [23, see for a review: 2]. If guilt and shame are indeed involved in child and adolescent psychopathology, one would expect that these emotions will be particularly prominent in clinically referred youth. The few studies that have been conducted in clinical settings so far [24, 25: Study 2] relied on populations that were ill-described in terms of psychopathology and were limited in terms of sample size (*N*’s being 41 and 20, respectively). The results of these investigations were also quite mixed, which is not particularly surprising given earlier findings that relations between guilt and shame and psychopathological symptoms may critically depend on the type of psychopathology under study. With these drawbacks in mind, the present research project was conducted to further explore the links between the self-conscious emotions of guilt and shame and psychopathology in clinical youth.

A sample of 1000 children and adolescents who were referred to an outpatient treatment center for mental health problems were included in the study. The key instrument of this study was the Achenbach System of Empirically Based Assessment (ASEBA [26, 27]), which appeared preeminently suitable as it not only includes (a) three items for measuring self-conscious emotions, namely item 26 “Lacks guilt”, item 52 “Feels very guilty”, and item 71 “Self-conscious, easily ashamed”, but also (b) assesses symptoms of most common internalizing (i.e., anxiety problems and affective problems) and externalizing (i.e., attention-deficit/hyperactivity problems, oppositional defiant problems, conduct problems) problems, in keeping with the Diagnostic and Statistical Manual of Mental disorders (DSM) classification system [28]. As the ASEBA instrument can be completed by parents, teachers, and youths themselves, it provided the unique opportunity to study intensity levels of self-conscious emotions and their relations to various types of psychopathology in clinically referred children and adolescents, using a multi-informant approach.

We expected that intensity levels for lack of guilt, guilt, and shame in these clinically referred youths would be higher than those reported for non-clinical children and adolescents in the Dutch manual of the ASEBA instrument [29–31]. In addition, it was predicted that high intensity levels of shame would be associated with higher levels of internalizing as well as externalizing symptoms, high intensity levels of guilt would be associated with higher levels of internalizing symptoms, whereas low levels of guilt and especially lack of guilt would be associated with higher levels of externalizing symptoms. These predictions were examined in two ways: first by comparing lack of guilt, guilt, and shame levels among children and adolescents with various types of clinical diagnoses, and second by exploring the relationships between lack of guilt, guilt, and shame and youth’s scores on the main DSM-oriented internalizing and externalizing scales of the ASEBA instrument. Gender and age differences were also taken into account as previous research has indicated that these demographic variables have an impact on youth’s experiences of self-conscious emotions. That is, girls tend to display somewhat higher levels of guilt and shame as compared to boys [32], and it is also assumed that there is a clear age-related progression of children’s understanding and experience of these self-conscious emotions [33–35].

## Method

### Participants and procedure

Participants were children and adolescents who were consecutively referred to RIAGG Maastricht between 2007 and 2013 and for which parents and teachers had completed the ASEBA instrument as part of the standard intake procedure. RIAGG Maastricht (currently Virenze-RIAGG Maastricht) is an outpatient diagnostics and treatment facility for youth with mental health problems. Upon referral to this facility, all children and adolescents were subjected to an extensive diagnostic procedure that followed the longitudinal-expert-all data (LEAD) principle [36]. More specifically, the longitudinal aspect of the diagnostic procedure included the repeated revision of (prior) diagnoses as new information became available during intake, diagnostics, and treatment. The experts were all licensed psychologists and psychiatrists who were professionally trained in classifying mental disorders in youth, with or without a (semi-) structured interview. Data from multiple sources (i.e., play sessions and/or interviews with the child, interviews with parents and the teacher, psychological assessment, psychiatric examinations, observations at the facility, at home, and/or at school) were used to establish a DSM-IV-based clinical diagnosis that was the primary focus of treatment. The study was officially approved by the Ethical Committee of Psychology (ECP) at Maastricht University.

A team of six psychology master students started to systematically search the files of the youth that had been referred to our clinical facility to retrieve the following data: (1) the ASEBA instrument as completed by parents and teachers (which were almost always available as these scales were an integral part of the intake assessment) and eventually by youths themselves (in case they were 11 years or older and capable/willing to complete the measure), (2) socio-demographic variables such as gender, age, and IQ (if available), and (3) the primary clinical diagnosis as established according to the LEAD principle (see above). The students began with the files of youths referred in 2013 and worked backwards in time to 2007 till they retrieved 1000 children and adolescents for which at least the parent and teacher ASEBA instrument data were completely available. Characteristics of these youths are displayed in Table 1. As can be seen, the sample consisted of 652 boys and 348 girls, had a mean age of 10.60 years (SD = 3.30), and included more younger (i.e., 4- to 11-year-olds) than older youths (i.e., 12- to 18-year-olds), with *n*’s being 617 versus 383, respectively. Four-hundred-and-forty-six of these participants (almost 95 % of the 11- to 18-year-olds) had completed the self-report version of the ASEBA instrument. IQ data were available for 437 children and adolescents: the mean IQ was 98.32 (range 59–150). Attention-Deficit/Hyperactivity Disorder (ADHD) and pervasive developmental disorders (including autistic disorder, Asperger disorder, and pervasive developmental disorder-not otherwise specified) were the most frequent primary clinical diagnoses, together representing 59.3 % of the total sample. Other common diagnoses were parent–child relationship problem, anxiety disorders (including obsessive–compulsive disorder and post-traumatic stress disorder), depressive disorders (major depressive disorder, dysthymic disorder), disruptive behavior disorders (oppositional defiant disorder, conduct disorder), adjustment disorder, and identity/personality problems. Only 11 children and adolescents (1.1 %) did not receive a clinical diagnosis.

**Table 1:** Characteristics of the clinically referred youths who were included in this study

	*N*, *M* (SD) or %
Gender	
Boys	652
Girls	348
Age	10.60 (3.30)
4–11 years	617
12–18 years	383
11–18 years (YSR)	446
IQ^a^	98.32 (16.45)
Primary clinical diagnosis	
Depressive disorders	56
Anxiety disorders	80
Pervasive developmental disorders	227
Identity/personality problems	27
ADHD	366
Parent–child relationship problem	88
Disruptive behavior disorders	50
Adjustment disorder	38
Learning disorders	16
Reactive attachment disorder	14
Eating disorders	4
Somatoform disorders	3
Other disorders	20
No diagnosis	11
ASEBA percentage in clinical range^b^	
CBCL	57.3
TRF	37.2
YSR	37.7
CBCL or TRF	69.9
CBCL or TRF or YSR^c^	74.4

*IQ* Intelligence Quotient, *ADHD* Attention-Deficit/Hyperactivity Disorder, *ASEBA* Achenbach System Of Empirically Based Assessment, *CBCL* Child Behavior Checklist, *TRF* Teacher Report Form, *YSR* Youth Self Report

^a^Based on *N* = 437

^b^Number of participants scoring in the clinical range on at least one DSM-oriented scale (excluding somatic problems)

^c^ *N* = 446

### Instrument

As noted earlier, the *ASEBA*—*school*-*age version* [26, 27] was the main assessment instrument used in this study. The ASEBA is a widely employed measure for assessing emotional and behavioral problems in youth. It adopts a multi-informant approach as there are similar forms to be completed by parents (the Child Behavior Checklist), teachers (the Teacher Report Form), and—from 11 years onwards—children themselves (the Youth Self-Report). In total, the instrument contains 112 problem items for which each informant has to rate to what extent they are applicable to the child (0 = not true, 1 = somewhat true, and 2 = very true). Items can be combined to DSM-oriented scales, which were preferred over the empirically derived syndrome scales given the fact that the former appear to have (somewhat) greater clinical utility [37–39]. We only included those DSM-oriented scales that were most characteristic for the clinically referred children and adolescents in this study, namely affective problems, anxiety problems, attention-deficit/hyperactivity problems, oppositional defiant problems, and conduct problems. Furthermore, of special interest were the three ASEBA items referring to youth’s self-conscious emotions: item 26 “Lacks guilt”, item 52 “Feels very guilty”, and item 71 “Self-conscious, easily ashamed”^1^ representing, respectively, the concepts of lack of guilt, guilt, and shame. Research has indicated that the ASEBA provides a reliable and valid indicator of psychopathology in youth [26] and this is also true for the Dutch version of the instrument [29–31, 40].

### Statistical analyses

First, *t* tests for independent samples were conducted to compare intensity levels for lack of guilt, guilt, and shame in these clinically referred youths with those reported for non-clinical children and adolescents in the Dutch manual of the ASEBA instrument [29–31]. Second, to explore gender and age effects in intensity levels for lack of guilt, guilt, and shame, a series of 2 (gender: boys vs. girls) × 2 (age groups: 4- to 11-year-olds vs. 12- to 18-year-olds) analyses of variance (ANOVAs) were conducted on the CBCL and TRF scores of items 26, 52, and 71. As the YSR was only completed by youth aged from 11 years onwards, one-way ANOVAs were used to only evaluate gender differences in scores on items 26, 52, and 71 of this scale. Third, ANOVAs with gender and age as covariates (ANCOVAs) were carried out to compare lack of guilt, guilt, and shame levels among children and adolescents with various types of clinical diagnoses. Only those clinical diagnoses were included in these analyses for which a considerable number of youth (i.e., >2 %) were present in this sample and for which clear predictions regarding levels of lack of guilt, guilt, and shame could be made.^2^ Fourth and finally, to explore the relationship between lack of guilt, guilt, and shame, on the one hand, and youth’s scores on the main DSM-oriented internalizing and externalizing scales of the ASEBA instrument on the other hand, a series of ANCOVAs with again gender and age as covariates were conducted comparing groups of youth with low, medium, and high levels for lack of guilt, guilt, or shame on the ASEBA instrument with regard to their symptom level scores on the DSM-oriented scales of affective problems, anxiety problems, ADHD problems, oppositional defiant problems, and conduct problems. In each of the analyses, we not only controlled for gender and age but also for symptoms of comorbid psychopathology.^3^ Quite a large number of statistical tests were conducted for this study, which of course inflates the risk of type I error. However, our hypotheses were very specific, and therefore we chose to report differential *p* values (i.e., *p* < .05 and *p* < .001) instead of conducting a formal correction for multiple testing. Note that all the analyses showing an effect at *p* < .001 would certainly survive such a statistical (e.g., Bonferroni) correction. Further, effect sizes were reported to give a clear impression of the actual magnitude of the observed effects.

## Results

### Intensity levels of self-conscious emotions

Mean intensity levels (standard deviations) for lack of guilt, guilt, and shame as measured by items 26, 52, and 71 of various ASEBA scales in the present sample are shown in Tables 2, 3, and 4. Independent samples *t* tests indicated that these clinically referred youth generally displayed higher levels of lack of guilt, guilt, and shame as reported by parents, teachers, and children themselves than a non-clinical comparison group of youth recruited from the general Dutch population. Only in two cases (TRF item 52 “Feels very guilty” in 4- to 11-year-old boys and YSR item 52 “Feels very guilty” in 11- to 18-year-old boys; see Table 3), the difference was not significant. Note also that the percentages of endorsement in the extreme (i.e., very true) category in this clinical sample were on average 5.5 times (range 1.5–12.8) larger for item 26 “Lacks guilt”, 10.6 times (range 1.7–33) larger for item 52 “Feels very guilty”, and 5.0 times (range 1.7–8.3) larger for item 71 “Self-conscious, easily ashamed” than in the non-clinical comparison group.

**Table 2:** Frequencies of positive endorsement of ASEBA item 26 “Lacks guilt” and mean scores (standard deviations) as found in the present sample

	N	*M* (SD) Present sample	% endorsing very true	*M* (SD) non-clinical comparison group^1^	% endorsing very true
CBCL item 26 “Lacks guilt”					
Boys 4–11 years	409	0.69 (0.80)_a_	21.3	0.24 (0.49)**	2.6
Boys 12–18 years	243	0.71 (0.80)_a_	21.8	0.23 (0.50)**	3.6
Girls 4–11 years	208	0.43 (0.63)_b_	7.3	0.19 (0.44)**	2.0
Girls 12–18 years	140	0.69 (0.80)_a_	20.7	0.18 (0.47)**	3.7
TRF item 26 “Lacks guilt”					
Boys 4–11 years	409	0.60 (0.76)_a_	16.9	0.24 (0.53)**	5.2
Boys 12–18 years	243	0.56 (0.77)_a_	17.3	0.18 (0.44)**	2.5
Girls 4–11 years	208	0.35 (0.66)_b_	10.2	0.08 (0.31)**	0.8
Girls 12–18 years	140	0.39 (0.67)_b_	10.0	0.11 (0.38)**	2.1
YSR item 26 “Lacks guilt”					
Boys 11–18 years	285	0.68 (0.75)_a_	17.2	0.56 (0.68)*	10.9
Girls 11–18 years	161	0.59 (0.73)_a_	14.3	0.45 (0.66)*	9.6

For reasons of comparison, previously reported normative mean scores (standard deviations) of Dutch non-clinical youths are also shown

*ASEBA* Achenbach System of Empirically Based Assessment, *CBCL* Child Behavior Checklist, *TRF* Teacher Report Form, *YSR* Youth Self Report

^1^As reported in the Dutch manuals of CBCL/TRF/YSR [29–31]. For each item, within-column means not sharing similar subscripts differ at *p* < .05

* Significant difference with the present sample at *p* < .05

** Significant difference with the present sample at *p* < .001

**Table 3:** Frequencies of positive endorsement of ASEBA item 52 “Very guilty” and mean scores (standard deviations) as found in the present sample

	N	*M* (SD) present sample	% endorsing very true	*M* (SD) non-clinical comparison group^1^	% endorsing very true
CBCL item 52 “Feels very guilty”					
Boys 4–11 years	409	0.21 (0.47)_a_	2.5	0.05 (0.23)**	0.2
Boys 12–18 years	243	0.35 (0.57)_b_	4.9	0.07 (0.28)**	0.5
Girls 4–11 years	208	0.31 (0.57)_b_	5.3	0.07 (0.27)**	0.3
Girls 12–18 years	140	0.44 (0.65)_b_	8.6	0.12 (0.35)**	0.9
TRF item 52 “Feels very guilty”					
Boys 4–11 years	409	0.08 (0.30)_a_	0.7	0.05 (0.24)	0.4
Boys 12–18 years	243	0.21 (0.48)_b_	3.3	0.03 (0.17)**	0.0
Girls 4–11 years	208	0.10 (0.33)_a_	1.0	0.04 (0.21)*	0.0
Girls 12–18 years	140	0.31 (0.62)_b_	8.6	0.09 (0.34)**	1.5
YSR item 52 “Feels very guilty”					
Boys 11–18 years	285	0.33 (0.59)_a_	6.3	0.27 (0.53)	3.8
Girls 11–18 years	161	0.61 (0.73)_b_	14.9	0.33 (0.55)**	3.8

For reasons of comparison, previously reported normative mean scores (standard deviations) of Dutch non-clinical youths are also shown

*ASEBA* Achenbach System of Empirically Based Assessment, *CBCL* Child Behavior Checklist, *TRF* Teacher Report Form, *YSR* Youth Self Report

^1^As reported in the Dutch manuals of CBCL/TRF/YSR [29–31]. For each item, within-column means not sharing similar subscripts differ at *p* < .05

* Significant difference with the present sample at *p* < .05

** Significant difference with the present sample at *p* < .001

**Table 4:** Frequencies of positive endorsement of ASEBA item 71 “Self-conscious, easily ashamed” and mean scores (standard deviations) as found in the present sample

	N	*M* (SD) present sample	% endorsing very true	*M* (SD) non-clinical comparison group^1^	% endorsing very true
CBCL item 71 “Self-conscious, easily ashamed”					
Boys 4–11 years	409	0.56 (0.69)_a_	11.5	0.27 (0.51)**	3.1
Boys 12–18 years	243	0.65 (0.72)_a_	14.8	0.27 (0.52)**	3.9
Girls 4–11 years	208	0.60 (0.72)_a_	14.1	0.25 (0.47)**	1.7
Girls 12–18 years	140	0.87 (0.76)_b_	22.9	0.32 (0.53)**	3.3
TRF item 71 “Self-conscious, easily ashamed”					
Boys 4–11 years	409	0.44 (0.62)_a_	7.1	0.29 (0.51)**	2.5
Boys 12–18 years	243	0.53 (0.72)_a_	13.2	0.20 (0.44)**	1.6
Girls 4–11 years	208	0.50 (0.68)_a_	10.2	0.24 (0.48)**	2.5
Girls 12–18 years	140	0.75 (0.78)_b_	20.7	0.27 (0.50)**	2.7
YSR item 71 “Self-conscious, easily ashamed”					
Boys 11–18 years	285	0.57 (0.66)_a_	9.5	0.42 (0.59)*	5.5
Girls 11–18 years	161	0.94 (0.72)_b_	23.0	0.62 (0.67)**	10.4

For reasons of comparison, previously reported normative mean scores (standard deviations) of Dutch non-clinical youths are also shown

*ASEBA* Achenbach System of Empirically Based Assessment, *CBCL* Child Behavior Checklist, *TRF* Teacher Report Form, *YSR* Youth Self Report

^1^As reported in the Dutch manuals of CBCL/TRF/YSR [29–31]. For each item, within-column means not sharing similar subscripts differ at *p* < .05

* Significant difference with the present sample at *p* < .05

** Significant difference with the present sample at *p* < .001

Paired samples *t* tests revealed that in general intensity levels for lack of guilt, guilt, and shame were significantly lower when reported by teachers (means on TRF items 26, 52, and 71 being .50, SD = .74, .15, SD = .42, and .52, SD = .69) than when reported by parents (means on CBCL items 26, 52, and 71 being .64, SD = .77, .30, SD = .55, and .64, SD = .72) and children themselves (means on YSR items 26, 52, and 71 being .65, SD = .74, .43, SD = .66, and .71, SD = .70) [all *t*(445/999)’s ≥2.56, *p* < .05]. Further, cross-informant correlations were rather small, and ranged between .12 (*p* < .05) and .21 (*p* < .001) for lack of guilt, between .16 (*p* < .01) and .29 (*p* < .001) for guilt, and between .30 (*p* < .001) and .33 (*p* < .001) for shame.

### Gender and age effects

The series of ANOVAs evaluating gender and age effects for the intensity of various types of self-conscious emotions yielded the following significant findings. First, with the exception of YSR item 26 “Lacks guilt”, all analyses revealed a significant effect of gender [*F*(1,996/1,444)’s ranging between 5.28 and 30.67, *p*’s < .05, *η*^2^’s between .01 and .07]. As can be seen in Tables 2, 3, and 4, boys displayed higher intensity levels of lack of guilt as reported by parents and teachers, whereas girls exhibited higher intensity levels of guilt and shame as reported by parents, teachers, and children themselves. Second, on TRF item 26 “Lacks guilt” no significant effect of age group was found, but for all the other parent (CBCL)- and teacher (TRF)-reported self-conscious emotions such an effect did emerge [*F*(1,996/1,444)’s ranging between 6.88 and 36.44, *p*’s < .01, *η*^2^’s between .01 and .04]. The data indicated that parents and teachers reported higher levels of lack of guilt, guilt, and shame for older youth than for their younger counterparts (see Tables 2, 3, 4). Third, only in the case of CBCL item 26 “Lacks guilt”, a significant interaction effect of gender and age was found [*F*(1,996) = 5.59, *p* < .01, *η*^2^ = .01]. As can be seen in Table 2, younger and older boys displayed similar levels for lack of guilt, whereas younger girls exhibited significantly lower lack of guilt scores than their older counterparts.

### Lack of guilt, guilt, and shame and clinical diagnoses

Table 5 shows CBCL, TRF, and YSR lack of guilt, guilt, and shame scores of children and adolescents with various types of clinical diagnoses. A series of ANCOVAs (with gender and age as covariates) indicated that lack of guilt, guilt, and shame levels were significantly different among various disorders [*F*(6,885/6,389)’s ranging between 2.17 and 13.09, *p*’s < .05, *η*^2^’s between .02 and .08]. Post hoc comparisons revealed that findings were generally in keeping with our predictions. That is, lack of guilt scores was highest among children and adolescents with disruptive behavior disorders (CBCL, TRF, and YSR) and youth diagnosed with parent–child relationship problem (CBCL), whereas fairly low lack of guilt scores was found for children and adolescents with anxiety disorders (CBCL). Further, for guilt, fairly high scores were observed for youth with depressive disorders (CBCL, TRF, and YSR), anxiety disorders (TRF, YSR), and identity/personality problems (CBCL), while relatively low guilt scores were found for youth with ADHD (CBCL, TRF, and YSR), pervasive developmental disorders (TRF), parent–child relationship problem (TRF), and disruptive behavior disorders (TRF). Finally, for shame, the highest scores were observed for pervasive developmental disorders (CBCL) and anxiety disorders (TRF and YSR), whereas fairly low scores were noted for ADHD (CBCL, TRF, and YSR) and disruptive behavior disorders (CBCL and TRF).

**Table 5:** Mean lack of guilt, guilt, and shame scores (standard errors) as measured by various ASEBA scales for children and adolescents with various clinical diagnoses

	Item 26 “Lacks guilt”		
	CBCL	TRF	YSR
Primary DSM classification			
Depressive disorders (*n* = 56/42^†^)	0.65 (0.10)_a_	0.32 (0.10)_a_	0.58 (0.12)_a_
Anxiety disorders (*n* = 80/52)	0.37 (0.09)_b_	0.36 (0.08)_a_	0.49 (0.10)_a_
Pervasive developmental disorders (*n* = 227/94)	0.68 (0.05)_a_	0.45 (0.05)_a_	0.60 (0.08)_a_
Identity/personality problems (*n* = 27/25)	0.76 (0.15)_ac_	0.56 (0.14)_a_	0.49 (0.15)_a_
ADHD (*n* = 366/121)	0.56 (0.04)_a_	0.55 (0.04)_a_	0.69 (0.07)_a_
Parent–child relationship problem (*n* = 88/38)	0.86 (0.08)_c_	0.54 (0.08)_a_	0.71 (0.12)_ab_
Disruptive behavior disorders (*n* = 50/26)	1.00 (0.11)_c_	1.01 (0.10)_b_	1.07 (0.15)_b_
*F*(6,885/6,389)	5.58**	5.61**	2.17*
*η* ^2^	.04	.03	.03

	Item 52 “Feels very guilty”		
	CBCL	TRF	YSR
Primary DSM classification			
Depressive disorders (*n* = 56/42)	0.47 (0.07)_a_	0.29 (0.05)_a_	0.74 (0.10)_a_
Anxiety disorders (*n* = 80/52)	0.39 (0.06)_ab_	0.36 (0.05)_a_	0.66 (0.09)_a_
Pervasive developmental disorders (*n* = 227/94)	0.31 (0.03)_bc_	0.12 (0.03)_b_	0.30 (0.07)_b_
Identity/personality problems (*n* = 27/25)	0.55 (0.11)_a_	0.14 (0.08)_ab_	0.52 (0.13)_ab_
ADHD (*n* = 366/121)	0.24 (0.03)_c_	0.10 (0.02)_b_	0.29 (0.06)_b_
Parent–child relationship problem (*n* = 88/38)	0.32 (0.06)_bc_	0.10 (0.04)_b_	0.47 (0.10)_ab_
Disruptive behavior disorders (*n* = 50/26)	0.27 (0.08)_bc_	0.11 (0.06)_b_	0.56 (0.13)_ab_
*F*(6,885/6,389)	2.60*	5.96**	4.32**
*η* ^2^	.02	.04	.06

	Item 52 “Self-conscious, easily ashamed”		
	CBCL	TRF	YSR
Primary DSM classification			
Depressive disorders (*n* = 56/42)	0.77 (0.10)_ab_	0.53 (0.09)_a_	0.90 (0.10)_ab_
Anxiety disorders (*n* = 80/52)	0.80 (0.08)_ab_	0.85 (0.07)_b_	1.13 (0.09)_a_
Pervasive developmental disorders (*n* = 227/94)	0.88 (0.05)_a_	0.73 (0.04)_b_	0.66 (0.07)_bc_
Identity/personality problems (*n* = 27/25)	0.84 (0.14)_ab_	0.52 (0.13)_a_	0.76 (0.14)_b_
ADHD (*n* = 366/121)	0.44 (0.04)_b_	0.35 (0.03)_c_	0.52 (0.06)_c_
Parent–child relationship problem (*n* = 88/38)	0.63 (0.07)_ab_	0.45 (0.07)_ac_	0.66 (0.11)_bc_
Disruptive behavior disorders (*n* = 50/26)	0.45 (0.10)_b_	0.24 (0.09)_c_	0.67 (0.13)_bc_
*F*(6,885/6,389)	11.65**	13.09**	5.58**
*η* ^2^	.07	.08	.08

*ASEBA* Achenbach System of Empirically Based Assessment, *CBCL* Child Behavior Checklist, *TRF* Teacher Report Form, *YSR* Youth Self Report

^†^Left numbers pertain to *n* for CBCL/TRF data, right numbers pertain to *n* for YSR data. All analyses were conducted while controlling for age and gender. Within-column means not sharing similar subscripts differ at *p* < .05

### Relations between lack of guilt, guilt, and shame and DSM-oriented ASEBA scales

To further explore the relation between self-conscious emotions and psychopathology, ANCOVAs (controlling for gender, age, and comorbid psychopathology^4^) were performed to compare groups of youth with varying levels of lack of guilt, guilt, and shame with regard to their scores on the main DSM-oriented scales of the ASEBA instrument. Results indicated that lack of guilt was mainly related to the ASEBA scales of conduct problems (CBCL, TRF, and YSR), oppositional defiant problems (CBCL and TRF), and ADHD problems (TRF) [*F*(2,991/2,437)’s ranging between 7.52 and 36.58, *p*’s < .05, *η*^2^’s between .03 and .07]. As shown in Table 6, youth scoring ‘somewhat true’ or ‘very true’ on item 26 “Lacks guilt” displayed significantly higher levels of these externalizing symptoms than youth scoring ‘not true’ on this item. For some scales (i.e., CBCL oppositional defiant problems, CBCL conduct problems, and TRF conduct problems), the difference in problem levels between youth scoring ‘somewhat true’ and those scoring ‘very true’ was also significant, pointing at a clear-cut linear relation between lack of guilt and these types of psychopathology.

**Table 6:** Mean scores (standard errors) on various ASEBA DSM-oriented scales of clinically referred youths with varying levels of lack of guilt reported by parents, teachers, and youths themselves

	CBCL item 26 “Lacks guilt”				
	Not true (*n* = 541)	Somewhat true (*n* = 275)	Very true (*n* = 184)	*F* (2,991)	*η* ^2^
CBCL DSM-oriented scale					
Affective problems	4.95 (0.14)	4.84 (0.19)	5.24 (0.25)	0.87	.00
Anxiety problems	3.25 (0.11)	3.33 (0.14)	3.10 (0.19)	0.49	.00
ADHD problems	7.30 (0.15)	7.36 (0.19)	7.74 (0.26)	1.03	.00
Oppositional defiant problems	3.85 (0.08)_a_	4.71 (0.11)_b_	4.99 (0.15)_c_	27.37**	.05
Conduct problems	3.39 (0.11)_a_	4.13 (0.15)_b_	5.49 (0.20)_c_	36.58**	.07

	TRF item 26 “Lacks guilt”				
	Not true (*n* = 637)	Somewhat true (*n* = 217)	Very true (*n* = 146)	*F*(2,991)	*η* ^2^
TRF DSM-oriented scale					
Affective problems	3.19 (0.10)	3.44 (0.17)	3.49 (0.22)	0.99	.00
Anxiety problems	2.46 (0.09)	2.26 (0.14)	2.23 (0.19)	0.82	.00
ADHD problems	10.15 (0.25)_a_	13.01 (0.40)_b_	12.54 (0.54)_b_	17.63**	.03
Oppositional defiant problems	2.45 (0.07)_a_	3.18 (0.12)_b_	3.42 (0.15)_b_	19.27**	.04
Conduct problems	2.72 (0.11)_a_	3.41 (0.17)_b_	4.79 (0.23)_c_	30.03**	.06

	YSR item 26 “Lacks guilt”				
	Not true (*n* = 228)	Somewhat true (*n* = 146)	Very true (*n* = 72)	*F*(2,437)	*η* ^2^
YSR DSM-oriented scale					
Affective problems	6.06 (0.24)	5.74 (0.29)	6.12 (0.42)	0.45	.00
Anxiety problems	3.29 (0.14)	3.11 (0.17)	3.43 (0.25)	0.65	.00
ADHD problems	6.56 (0.19)	7.22 (0.24)	6.74 (0.34)	2.23	.01
Oppositional defiant problems	3.10 (0.10)	3.41 (0.12)	3.08 (0.17)	2.14	.01
Conduct problems	3.55 (0.15)_a_	4.07 (0.18)_b_	4.66 (0.25)_b_	7.52*	.03

*ASEBA* Achenbach System of Empirically Based Assessment, *CBCL* Child Behavior Checklist, *TRF* Teacher Report Form, *YSR* Youth Self Report

All analyses were conducted while controlling for age, gender, and symptoms of comorbid psychopathology. Within-row means not sharing similar subscripts differ at *p* < .05. * p < .05, ** p < .001

Guilt and shame were consistently related to the ASEBA scales of affective problems and anxiety problems [*F*(2,991/2,437)’s ranging between 4.02 and 96.87, *p*’s < .05, *η*^2^’s between .01 and .17]. As can be seen in Tables 7 and 8, youth scoring ‘very true’ on item 52 “Feels very guilty” and item 71 “Self-conscious, feeling ashamed” exhibited significantly higher levels of these internalizing symptoms than youth scoring ‘not true’ on these items. Youth scoring ‘somewhat true’ on these items generally displayed intermediate symptom levels, although differences with the extreme groups were not always significant.

**Table 7:** Mean scores (standard errors) on various ASEBA DSM-oriented scales of clinically referred youths with varying levels of guilt reported by parents, teachers, and youth themselves

	CBCL item 52 “Feels very guilty”				
	Not true (*n* = 749)	Somewhat true (*n* = 206)	Very true (*n* = 45)	*F*(2,991)	*η* ^2^
CBCL DSM-oriented scale					
Affective problems	4.66 (0.11)_a_	5.86 (0.22)_b_	6.16 (0.46)_b_	13.80**	.03
Anxiety problems	2.87 (0.08)_a_	4.24 (0.16)_b_	4.91 (0.34)_b_	38.17**	.07
ADHD problems	7.34 (0.12)	7.58 (0.23)	7.47 (0.48)	0.41	.00
Oppositional defiant problems	4.40 (0.07)_a_	3.96 (0.14)_b_	4.16 (0.29)_ab_	3.99*	.01
Conduct problems	3.91 (0.10)	4.19 (0.19)	4.13 (0.40)	0.82	.00

	TRF item 52 “Feels very guilty”				
	Not true (*n* = 877)	Somewhat true (*n* = 98)	Very true (*n* = 25)	*F*(2,991)	*η* ^2^
TRF DSM-oriented scale					
Affective problems	3.18 (0.08)_a_	3.62 (0.24)_a_	5.60 (0.48)_b_	12.81**	.03
Anxiety problems	2.18 (0.07)_a_	3.77 (0.20)_b_	4.03 (0.40)_b_	35.31**	.07
ADHD problems	11.06 (0.20)	11.63 (0.60)	11.37 (1.21)	0.41	.00
Oppositional defiant problems	2.76 (0.06)	2.74 (0.17)	2.22 (0.35)	1.18	.00
Conduct problems	3.18 (0.09)	3.02 (0.26)	3.27 (0.53)	0.20	.00

	YSR item 52 “Feels very guilty”				
	Not true (*n* = 294)	Somewhat true (*n* = 110)	Very true (*n* = 42)	*F*(2,437)	*η* ^2^
YSR DSM-oriented scale					
Affective problems	5.55 (0.21)_a_	6.28 (0.34)_ab_	7.99 (0.57)_b_	7.49*	.03
Anxiety problems	2.72 (0.12)_a_	4.07 (0.19)_b_	4.82 (0.32)_b_	26.69**	.11
ADHD problems	6.72 (0.18)	6.76 (0.29)	7.56 (0.48)	1.34	.01
Oppositional defiant problems	3.18 (0.09)	3.32 (0.15)	3.06 (0.25)	0.58	.00
Conduct problems	3.73 (0.14)	4.31 (0.22)	4.04 (0.37)	2.31	.00

*ASEBA* Achenbach System of Empirically Based Assessment, *CBCL* Child Behavior Checklist, *TRF* Teacher Report Form, *YSR* Youth Self Report

All analyses were conducted while controlling for age, gender, and symptoms of comorbid psychopathology. Within-row means not sharing similar subscripts differ at *p* < .05. * *p* < .05, ** *p* < .001

**Table 8:** Mean scores (standard errors) on various ASEBA DSM-oriented scales of clinically referred youths with varying levels of shame reported by parents, teachers, and youths themselves

	CBCL item 71 “Self-conscious, easily ashamed”				
	Not true (*n* = 509)	Somewhat true (*n* = 347)	Very true (*n* = 144)	*F*(2,991)	*η* ^2^
CBCL DSM-oriented scale					
Affective problems	4.72 (0.14)_a_	5.08 (0.16)_ab_	5.62 (0.28)_b_	4.02*	.01
Anxiety problems	2.43 (0.10)_a_	3.60 (0.11)_b_	5.25 (0.18)_c_	93.98**	.16
ADHD problems	7.74 (0.15)_a_	7.22 (0.17)_b_	6.61 (0.28)_b_	6.14*	.01
Oppositional defiant problems	4.29 (0.09)	4.36 (0.10)	4.20 (0.18)	0.38	.00
Conduct problems	3.85 (0.12)	4.00 (0.14)	4.38 (0.24)	1.82	.00

	TRF item 71 “Self-conscious, easily ashamed”				
	Not true (*n* = 595)	Somewhat true (*n* = 294)	Very true (*n* = 111)	*F*(2,991)	*η* ^2^
TRF DSM-oriented scale					
Affective problems	2.83 (0.10)_a_	3.74 (0.14)_b_	4.52 (0.24)_c_	22.23**	.05
Anxiety problems	1.72 (0.08)_a_	2.94 (0.11)_b_	4.47 (0.19)_c_	96.87**	.16
ADHD problems	11.98 (0.25)_a_	10.12 (0.34)_b_	9.17 (0.61)_b_	12.16**	.02
Oppositional defiant problems	2.83 (0.07)	2.68 (0.10)	2.47 (0.18)	1.67	.00
Conduct problems	3.16 (0.11)	3.13 (0.15)	3.32 (0.27)	0.22	.00

	YSR item 71 “Self-conscious, easily ashamed”				
	Not true (*n* = 195)	Somewhat true (*n* = 187)	Very true (*n* = 64)	*F*(2,437)	*η* ^2^
YSR DSM-oriented scale					
Affective problems	5.47 (0.27)_a_	5.94 (0.26)_a_	7.54 (0.48)_b_	6.18*	.03
Anxiety problems	2.27 (0.14)_a_	3.69 (0.14)_b_	4.98 (0.25)_c_	46.12**	.17
ADHD problems	6.90 (0.23)	6.67 (0.21)	6.68 (0.41)	0.12	.00
Oppositional defiant problems	3.22 (0.12)	3.17 (0.11)	3.23 (0.21)	0.06	.00
Conduct problems	3.88 (0.17)	4.04 (0.16)	3.58 (0.31)	1.01	.00

*ASEBA* Achenbach System of Empirically Based Assessment, *CBCL* Child Behavior Checklist, *TRF* Teacher Report Form, *YSR* Youth Self Report

All analyses were conducted while controlling for age, gender, and symptoms of comorbid psychopathology. Within-row means not sharing similar subscripts differ at *p* < .05. * p < .05, ** p < .001

For guilt, an additional effect was found for CBCL-rated oppositional defiant problems [*F*(2,991) = 3.99, *p* < .05]: that is, youth for which parents endorsed item 52 “Feels very guilty” with ‘not true’ displayed higher levels of this type of externalizing symptoms than youth for which parents endorsed this item with ‘somewhat true’ (see Table 7). In the case of shame, relations with parent- and teacher-reported ADHD problems were found [*F*(2,991)’s being 6.14 and 12.16, respectively, both *p*’s < .05]. As shown in Table 8, youth for which parents and teachers endorsed item 71 “Self-conscious, feeling ashamed” with ‘not true’ exhibited higher levels of ADHD symptoms than youth for which parents endorsed this item with ‘somewhat true’ or ‘very true’.

## Discussion

Previous research has indicated that psychopathology in youth is clearly linked with dysregulations in self-conscious emotions. That is, internalizing problems are typically accompanied by high levels of both guilt and shame, whereas externalizing problems are accompanied by high levels of shame on the one hand, and low levels, or even lack, of guilt on the other hand [2]. However, with few exceptions [24, 25: Study 2], this conclusion has mainly been based on studies conducted in non-clinical populations of children and adolescents. With this in mind, the present study was carried out to explore the relationship between self-conscious emotions and psychopathology in a large sample of clinically referred youth. For this purpose, we used the ASEBA instrument completed by parents, teachers, and children themselves during the regular intake procedure, which not only provides information on the intensity levels of lack of guilt, guilt, and shame, but also measures symptoms of the most prevalent types of internalizing and externalizing problems in youth. A first conclusion that can be drawn from these data is that the intensity levels of lack of guilt, guilt, and shame in these clinically referred children and adolescents were indeed considerably higher than those reported for non-clinical youth in the Dutch manual of the ASEBA instrument [29–31]. More precisely, across all three informants (i.e., parents, teachers, and children themselves), these clinically referred youth displayed significantly higher lack of guilt, guilt, and shame scores, with the percentage of endorsement in the extreme category of various types of self-conscious emotions being on average 5.0–10.6 times larger than in the non-clinical normative sample. Altogether, these results confirm the idea that youth suffering from various types of psychopathology indeed display clear dysregulations of self-conscious emotions.

The children and adolescents in the present sample had a variety of clinical diagnoses, of which ADHD, pervasive developmental disorders, parent–child relationship problem, anxiety disorders, depressive disorders, disruptive behavior disorders, adjustment disorder, and identity/personality problems were most prevalent. When comparing intensity levels of self-conscious emotions across groups of children and adolescents with various clinical diagnoses, an interesting pattern of results was found that was largely in keeping with the existing literature. That is, low levels of guilt and lack of guilt were associated with disruptive behavior disorders, high levels of guilt were related to internalizing problems such as anxiety and depressive disorders, whereas high levels of shame were most consistently linked to anxiety disorders. Apart from these main findings, a number of additional results were documented which deserve some further comment. First, lack of guilt was also high for youth with a parent–child relationship problem as the main clinical diagnosis, which is not that surprising as difficulties in the interaction between parents and child and associated family conflicts are often comorbid with externalizing problems such as oppositional defiant disorder and conduct disorder [41]. Second, children and adolescents with ADHD clearly showed low levels of guilt and shame. This is in keeping with a recent study in clinically referred adults showing that self-conscious emotions are less prominent in patients with this disorder than among patients with other psychiatric problems [42], and fits with the idea that youth with ADHD have a limited sense of self-awareness [43], which seems to be a prerequisite for experiencing self-conscious emotions [44]. Third, rather inconsistent findings emerged regarding the intensity of self-conscious emotions in youth with pervasive developmental disorders [45]: on the one hand, it was found that children and adolescents with this diagnosis displayed fairly high levels of shame, whereas on the other hand youth with this diagnosis exhibited relatively low levels of guilt. This disparity nevertheless matches with the social peculiarities of youth suffering from autism or related disorders. That is, these youngsters often do not feel at ease during social interactions and tend to show submissive and avoidance behavior [46], which might be reflected in high shame scores, but at the same time they lack interpersonal sensitivity and fail to recognize social transgressions [47], which might manifest in low guilt scores.

To further explore the relation between self-conscious emotions and psychopathology, analyses were conducted in which we compared groups of youth with varying levels of lack of guilt, guilt, and shame with regard to their scores on the main DSM-oriented scales of the ASEBA instrument. The results were largely in keeping with our predictions. To begin with, it was found that lack of guilt was positively associated with externalizing problems, which of course is in line with other research showing that “lack of remorse or guilt” is a prominent symptom in youth displaying aggressive and antisocial behavior [15]. Note also that this link was most consistently (i.e., across all three informants) found for conduct problems, which makes sense as lack of guilt normally is one of the symptoms defining the disorder [12]. In addition, the results indicated that guilt and shame were both positively associated with anxiety and depression problems, which confirms previous findings showing that these self-conscious emotions are more intense and frequent in children and adolescents with high levels of these types of internalizing symptoms [17, 19, 48, 49]. Finally, findings revealed that low intensity levels of guilt (parent report) and shame (parent and teacher report) were associated with relatively high levels of, respectively, oppositional defiant problems and ADHD problems. The former relation was certainly in line with the notion that guilt is low in individuals with externalizing problems [4, 15], while the latter link confirms the earlier mentioned results by Scheel et al. [42] who also noted fairly low levels of shame in adult patients with ADHD.

A remarkable result of the present study was the total absence of a positive link between shame and externalizing problems. While previous research has generally indicated that this self-conscious emotion is associated with higher levels of anger, aggression, and delinquency [2], neither the comparisons of shame levels across youth with various clinical diagnoses nor the analyses of the relation between shame and DSM-oriented ASEBA scales yielded evidence for such a relationship. If anything, the data pointed out that children and adolescents with disruptive behavior disorders (i.e., ODD and CD) were among those that—according to parents and teachers—displayed the lowest intensity levels of this self-conscious emotion. One way to bring these diverging findings into line would be to assume that the relation between shame and externalizing problems is non-linear in nature. That is, youth with low levels of shame display low levels of externalizing symptoms, those with intermediate levels of shame show higher levels of such symptoms (causing the positive relation in non-clinical samples), while youngsters with high levels of shame again exhibit low levels of externalizing problems. Such an inverted U-shape relationship could be tested in a mixed sample containing clinically referred as well as non-clinical children and adolescents. Another explanation has to do with the fact that previous studies largely neglected the issue of comorbid symptomatology. As also demonstrated in the present study, internalizing and externalizing problems frequently co-occur [50], and so it is quite likely that the relation between shame and externalizing is affected by the quite robust positive relation between shame and internalizing. A final account for the discrepancy between this study and previous investigations on the relation between shame and externalizing is more theoretical in nature. As noted by Muris and Meesters [2], the relation between feelings of shame and externalizing is probably mediated by a secondary appraisal process of blaming others, which apart from person-related factors originates from various context-related variables. It is possible that the outpatient clinic in which we conducted our research was visited by a rather specific population of youth that largely lacked this cognitive process.

The present study also yielded a number of additional findings that deserve brief discussion. First, the gender and age effects that were found were largely in line with what has been documented in the literature. That is, for girls, higher levels of guilt and shame were noted than for boys [32], showing that the former are more susceptible to these self-conscious emotions than the latter. Levels of lack of guilt, guilt, and shame were also found to be higher among 12- to 18-year-olds than among 4- to 11-year-olds, which corroborates the notion that there is an age-related progression in the experience of self-conscious emotions [33–35]. Further, this investigation relied on a multi-informant approach, which is considered as preferable when assessing psychopathology and emotions in youth [51]. Parents, teachers, and children themselves each tend to have their own perspective and this was illustrated in the present study by the rather small cross-informant correlations as found for lack of guilt, guilt, and shame. Interestingly, the pattern of results regarding the relations between these self-conscious emotions and youth’s psychopathology was quite similar for various informants, underlining that findings were quite robust.

The large sample size of clinically referred children and adolescents and the multi-informant approach are definitively strong features of this research. However, there are also a number of limitations that should be mentioned. One important shortcoming concerns the cross-sectional design of the study. As assessments were conducted by means of one measure and carried out within a short period of time, no conclusions can be drawn on the cause–effect relations between self-conscious emotions and psychopathology. From a developmental psychopathology perspective, it is interesting to view lack of guilt, guilt, and shame as antecedents of internalizing and externalizing problems in youth [2], but of course it is also possible that dysregulations of self-conscious emotions are an integral part of or emerge as a consequence of the child’s psychopathology. Another drawback is that dysregulations in youth’s self-conscious emotions were only measured with three single items taken from the ASEBA instrument. Although these items were clearly formulated and directly measured the intensity of lack of guilt, guilt, and shame in a straightforward way, no formal testing of the reliability and validity of this assessment has been conducted. Nevertheless, in the literature, good arguments can be found for the use of single-item scales [52]. Apart from the fact that single-item measures often do have acceptable validity, these also include the practical consideration of conducting an empirical study in a clinical population that is already subjected to a quite extensive psychological assessment (such as the children and adolescents in the present study). An additional demerit of our study concerns the fact that the relations between self-conscious emotions and psychopathology were partly investigated using data of one and the same instrument (i.e., the ASEBA), which of course raises the possibility that the observed significant effects were due to shared method variance. However, it seems highly unlikely that the specific pattern of results (i.e., lack of guilt, guilt, and shame were consistently associated with some types of symptoms but not with others) only emerged because constructs were assessed by means of one measure. Note further that we also related self-conscious emotions to clinical diagnoses, and these analyses by and large produced similar findings regarding the links between lack of guilt, guilt, and shame and psychopathology. A final limitation has to do with the fairly small effect sizes that were found in the analyses exploring the relationships between dysregulations in self-conscious emotions and psychopathology. One should bear in mind, however, that such associations are complex and that many other variables are involved. For instance, it is generally assumed that in the case of child and adolescent psychopathology the primary emotions of fear, sadness, and anger also play an important role [53, 54].

The present study showed that dysregulations of self-conscious emotions are quite prevalent among clinically referred youth. In addition, data consistently indicated that lack of guilt is especially implicated in oppositional defiant and conduct (i.e., externalizing) problems, while high levels of guilt and shame are preeminently associated with affective and anxiety (i.e., internalizing) problems. For most of these childhood problems effective, evidence-based treatments have been developed that primarily target on the correction of dysregulated basic emotions [55]. However, there is still a considerable proportion of the youth (i.e., up to 30 %) who do not respond adequately to these interventions [56]. For these cases, it may be relevant to also focus therapy on other pathogenic processes, such as the dysregulations of self-conscious emotions. Various authors have put forward that targeting this specific emotional aspect of psychopathology may yield better treatment results [57, 58], but in the upcoming years this notion needs to be investigated empirically.

## Footnotes

1 It is important to note that officially item 26 “Lacks guilt” and item 52 “Feels very guilty” are, respectively, part of the DSM-oriented scales ‘conduct problems’ and ‘affective problems’. To avoid the problem of shared covariance, these items were discarded when computing total scores for these DSM-oriented scales.

2 For this reason, children and adolescents with adjustment disorder were not included in these analyses, as this diagnosis is characterized by a mixture of internalizing and externalizing problems.

3 Thus, for example, the ANCOVA evaluating group differences for the ASEBA scale affective problems was controlled for gender, age, anxiety problems, ADHD problems, oppositional defiant problems, and conduct problems.

4 As expected, the ANCOVAs revealed significant effects of comorbid psychopathology: consistently large effect sizes were found between affective and anxiety problems and between oppositional defiant and conduct problems, but there were also significant comorbidity effects of internalizing on externalizing problems and vice versa.

## References

[1] Tangney JP, Tracy JL, Leary MR, Tangney JP (2012). Self-conscious emotions. Handbook of self and identity.

[2] Muris P, Meesters C (2012). Small or big in the eyes of the other. On the developmental psychopathology of self-conscious emotions as shame, guilt, and pride. Clin Child Fam Psychol Rev.

[3] Tracy JL, Robins RW (2004). Putting the self into self-conscious emotions: a theoretical model. Psychol Inq.

[4] Tangney JP, Wagner PE, Hill-Barlow D, Marschall DE, Gramzow R (1996). Relation of shame and guilt to constructive versus destructive responses to anger across the lifespan. J Personal Soc Psychol.

[5] Thompson RJ, Berenbaum H (2006). Shame reactions to everyday dilemmas are associated with depressive disorder. Cogn Ther Res.

[6] Andrews B, Brewin CR, Rose S, Kirk M (2000). Predicting PTSD symptoms in victims of violent crime: the role of shame, anger, and childhood abuse. J Abnorm Psychol.

[7] Fergus TA, Valentiner DP, McGrath P, Jencius S (2010). Shame- and guilt-proneness: relationships with anxiety disorder symptoms in a clinical sample. J Anxiety Disord.

[8] Troop NA, Allan S, Serpell L, Treasure JL (2008). Shame in women with a history of eating disorders. Eur Eat Disord Rev.

[9] Schoenleber M, Berenbaum H (2010). Shame aversion and shame proneness in Cluster C personality disorders. J Abnorm Psychol.

[10] Brown MZ, Linehan MM, Comtois KA, Murray A, Chapman AL (2009). Shame as a prospective predictor of self-inflicted injury in borderline personality disorder: a multi-modal analysis. Behav Res Ther.

[11] Dearing RL, Stuewig J, Tangney JP (2005). On the importance of distinguishing shame from guilt: relations to problematic alcohol and drug use. Addict Behav.

[12] American Psychiatric Association (2013). Diagnostic and statistical manual of mental disorders.

[13] Baumeister RF, Stillwell AM, Heatherton TF, Tangney JP, Fischer KW (1995). Interpersonal aspects of guilt: evidence from narrative studies. Self-conscious emotions: shame, guilt, embarrassment, and pride.

[14] Kochanska G, Gross JN, Lin MH, Nichols KE (2002). Guilt in young children: development, determinants, and relations with a broader system of standards. Child Dev.

[15] Stuewig J, Tangney JP, Heigel C, Harty L, McCloskey L (2010). Shaming, blaming, and maiming: functional links among the moral emotions, externalization of blame, and aggression. J Res Personal.

[16] Frick PJ, White SF (2008). Research review: the importance of callous-unemotional traits for developmental models of aggressive and antisocial behavior. J Child Psychol Psychiatry.

[17] Klasen F, Reissmann S, Voss C, Okello J (2015). The guiltless guilty: trauma-related guilt and psychopathology in former Ugandan child soldiers. Child Psychiatry Hum Dev.

[18] Mills RSL, Hastings PD, Serbin LA, Stack DM, Abela JRZ, Arbeau KA, Lall DIK (2015). Depressogenic thinking and shame proneness in the development of internalizing symptoms. Child Psychiatry Hum Dev.

[19] Muris P, Meesters C, Bouwman L, Notermans S (2015). Relations among behavioral inhibition, shame- and guilt-proneness, and anxiety disorders symptoms in non-clinical children. Child Psychiatry Hum Dev.

[20] Stuewig J, Tangney JP, Kendall S, Folk JB, Reinsmith Meyer C, Dearing RL (2015). Children’s proneness to shame and guilt predict risky and illegal behaviors in young adulthood. Child Psychiatry Hum Dev.

[21] Kochanska G, Barry RA, Jimenez NB, Hollatz AL, Woodard J (2009). Guilt and effortful control: two mechanisms that prevent disruptive developmental trajectories. J Personal Soc Psychol.

[22] Kim S, Thibodeau R, Jorgensen RS (2011). Shame, guilt, and depressive symptoms: a meta-analytic review. Psychol Bull.

[23] Ferguson TJ, Stegge H, Miller ER, Olsen ME (1999). Guilt, shame, and symptoms in children. Dev Psychol.

[24] Ferguson TJ, Stegge H, Eyre HL, Vollmer R, Ashbaker M (2000). Context effects and the (mal)adaptive nature of guilt and shame in children. Genet Soc Gen Psychol Monogr.

[25] Van Tijen N, Stegge H, Meerum Terwogt M, Van Panhuis N (2004). Anger, shame and guilt in children with externalizing problems: an imbalance of affects. Eur J Dev Psychol.

[26] Achenbach TM (2009). The Achenbach System of Empirically Based Assessment (ASEBA): development, findings, theory, and applications.

[27] Achenbach TM, Rescorla LA (2001). Manual for the ASEBA school-age forms & profiles.

[28] American Psychiatric Association (2000). Diagnostic and statistical manual of mental disorders, fourth edition-text revision.

[29] Verhulst FC, Van der Ende J, Koot HM (1997). Handleiding voor de Child Behavior Checklist/4-18 (Manual for the Child Behavior Checklist/4-18).

[30] Verhulst FC, Van der Ende J, Koot HM (1997b) Handleiding voor de Teacher Report Form (Manual for the Teacher Report Form). Department of Child and Youth Psychiatry, Erasmus University Rotterdam, Rotterdam

[31] Verhulst FC, Van der Ende J, Koot HM (1997). Handleiding voor de youth self-report (Manual for the Youth Self-Report).

[32] Else-Quest NM, Higgins A, Allison C, Morton LC (2012). Gender differences in self-conscious emotional experience: a meta-analysis. Psychol Bull.

[33] Berti AE, Garattoni C, Venturini B (2000). The understanding of sadness, guilt, and shame in 5-, 7-, and 9-year-old children. Genet Soc Gen Psychol Monogr.

[34] Leverato MC, Donati V (1999). Conceptual and lexical knowledge of shame in Italian children and adolescents. Int J Behav Dev.

[35] Olthof T, Schouten A, Kuiper H, Stegge H, Jennekens-Schinkel A (2000). Shame and guilt in children: differential situational antecedents and experiential correlates. Br J Dev Psychol.

[36] Spitzer RL (1983). Psychiatric diagnosis: are clinicians still necessary?. Compr Psychiatry.

[37] Ebesutani C, Bernstein A, Nakamura BJ, Chorpita BF, Higa-McMillan CK, Weisz JR, The Research Network on Youth Mental Health (2010). Concurrent validity of the Child Behavior Checklist DSM-oriented scales: correspondence with DSM diagnoses and comparison to syndrome scales. J Psychopath Behav Assess.

[38] Jensen PS, Saltzberg AD, Richters J, Watanabe HK, Roper M (1993). Scales, diagnoses, and child psychopathology. I. CBCL and DISC relationships. J Am Acad Child Adolesc Psychiatry.

[39] Kasius MC, Ferdinand RF, Van den Berg H, Verhulst FC (1997). Associations between different diagnostic approaches for child and adolescent psychopathology. J Child Psychol Psychiatry.

[40] Verhulst FC, Van der Ende J (2013). Handleiding ASEBA: Vragenlijsten voor leeftijden 6 tot en met 18 jaar—CBCL/6-18, YSR, TRF (Manual ASEBA: Questionnaires for ages 6 to 18 years—CBCL/6-18, YSR, TRF).

[41] Burt SA, Krueger RF, McGue M, Iacono W (2003). Parent-child conflict and the comorbidity among childhood externalizing disorders. Arch Gen Psychiatry.

[42] Scheel CN, Bender C, Tuschen-Caffier B, Brodführer A, Matthies S, Hermann C, Geisse EK, Svaldi J, Brakemeier EL, Philipsen A, Jacob GA (2014). Do patients with different mental disorders show specific aspects of shame. Psychiatry Res.

[43] Lou HC (2012). Paradigm shift in consciousness research: the child’s self-awareness and abnormalities in autism, ADHD, and schizophrenia. Acta Paediatr.

[44] Lewis M, Sullivan MW, Elliott AJ, Dweck CS (2005). The development of self-conscious emotions. Handbook of competence and motivation.

[45] Heerey EA, Keltner D, Capps LM (2003). Making sense of self-conscious emotion: linking theory of mind and emotion in children with autism. Emotion.

[46] Kuusikko S, Pollock-Wurman R, Jussila K, Carter AS, Mattila ML, Ebeling H, Pauls DL, Moilanen I (2008). Social anxiety in high-functioning children and adolescents with autism and Asperger syndrome. J Autism Dev Disord.

[47] Frith CD, Frith U (1999). Cognitive psychology—interacting minds—a biological basis. Science.

[48] Bennett DS, Sullivan MW, Lewis M (2010). Neglected children, shame-proneness, and depressive symptoms. Child Maltreat.

[49] De Rubeis S, Hollenstein T (2009). Individual differences in shame and depressive symptoms during early adolescence. Personal Individ Differ.

[50] Lilienfeld SO (2003). Comorbidity between and within childhood externalizing and internalizing disorders: reflections and directions. J Abnorm Child Psychol.

[51] Achenbach TM, McConaughy SH, Howell CT (1987). Child/adolescent behavioral and emotional problems: implications of cross-informant correlations for situational specificity. Psychol Bull.

[52] Gardner DG, Cummings LL, Dunham RB, Pierce JL (1998). Single-item versus multiple-item measurement scales: an empirical comparison. Educ Psychol Meas.

[53] Izard CE, Youngstrom EA, Fine SE, Mostow AJ, Trentacosta CJ, Cicchetti D, Cohen DJ (2006). Emotions and developmental psychopathology. Developmental psychopathology, theory and method.

[54] Muris P, Ollendick TH (2005). The role of temperament in the etiology of child psychopathology. Clin Child Fam Psychol Rev.

[55] Barrett PM, Ollendick TH (2004). Handbook of interventions that work with children and adolescents. Prevention and treatment.

[56] March JS (2009). The future of psychotherapy for mentally ill children and adolescents. J Child Psychol Psychiatry.

[57] Frick PJ, Ray JV, Thornton LC, Kahn RE (2014). Can callous-unemotional traits enhance the understanding, diagnosis, and treatment of serious conduct problems in children and adolescents? A comprehensive review. Psychol Bull.

[58] Gilbert P, Proctor S (2006). Compassionate mind training for people with high shame and self-criticism: a pilot study of a group therapy approach. Clin Psychol Psychother.

